# Application of Large Language Models (LLMs) to Geriatric Practice and Its Evaluation at 4 VA GRECCs

**DOI:** 10.1093/geroni/igaf122.1180

**Published:** 2025-12-31

**Authors:** Huai Cheng, Juliessa Pavon, Mo-Kyung Sin

## Abstract

LLMs application to clinical practice is growing fast. However, LLMs are less studied in geriatrics practice but are urgently needed. This symposium will address whether LLMs allocation to geriatric practice can be trusted via five approaches. 1) LLMs generated gender and race-biased outputs. We will demonstrate whether LLMs generated age-biased output by assessing their geriatric attitude evaluated by social workers. 2). LLMs passed USMLE and other examinations. We will demonstrate whether LLMs can pass geriatrics knowledge competence tests evaluated by geriatricians 3). LLMs performed well on clinical vignettes from different clinical disciplines. We will demonstrate whether LLMs can perform well on geriatrics 5M-based vignettes of older adults evaluated by clinical providers and trainees 4) LLMs reviewed and summarized clinical charts. We will demonstrate whether LLMs can review geriatrics and general medicine notes to extract Mobility (one of Geriatrics 5Ms) documentation evaluated by geriatricians 5). LLMs can generate deprescribing recommendations, tapering schedules, and patient education materials. We will demonstrate their accuracy, safety, and appropriateness compared to recommendations from a multidisciplinary team of pharmacists, geriatricians, and nurses. Specifically, this symposium will address the following topics: 1) Geriatric Attitude of ChatGPT4.o and Its Evaluation by Social Workers. 2) ChatGPT4.o Geriatrics Knowledge Competency and Its Evaluation by Geriatricians. 3) LLMs application to geriatrics 5Ms evaluated by clinical providers and trainees. 4) Using LLMs to Extract and Assess Mobility Documentation for Age-Friendly Health System evaluated by geriatricians. 5) Using LLMs to generate medication deprescribing recommendations compared to clinician-led deprescribing recommendations.

